# Twitter Predicts Citation Rates of Ecological Research

**DOI:** 10.1371/journal.pone.0166570

**Published:** 2016-11-11

**Authors:** Brandon K. Peoples, Stephen R. Midway, Dana Sackett, Abigail Lynch, Patrick B. Cooney

**Affiliations:** 1 Department of Forestry and Environmental Conservation, Clemson University, Clemson, South Carolina, United States of America; 2 Department of Oceanography and Coastal Sciences, Louisiana State University, Baton Rouge, Louisiana, United States of America; 3 School of Fisheries, Aquaculture, and Aquatic Sciences, Auburn University, Auburn, Alabama, United States of America; 4 National Climate Change and Wildlife Science Center, United States Geological Survey, Reston, Virginia, United States of America; 5 Smith-Root, Inc., Vancouver, Washington, United States of America; Max Planck Institute, GERMANY

## Abstract

The relationship between traditional metrics of research impact (e.g., number of citations) and alternative metrics (*altmetrics*) such as Twitter activity are of great interest, but remain imprecisely quantified. We used generalized linear mixed modeling to estimate the relative effects of Twitter activity, journal impact factor, and time since publication on Web of Science citation rates of 1,599 primary research articles from 20 ecology journals published from 2012–2014. We found a strong positive relationship between Twitter activity (i.e., the number of unique tweets about an article) and number of citations. Twitter activity was a more important predictor of citation rates than 5-year journal impact factor. Moreover, Twitter activity was not driven by journal impact factor; the ‘highest-impact’ journals were not necessarily the most discussed online. The effect of Twitter activity was only about a fifth as strong as time since publication; accounting for this confounding factor was critical for estimating the true effects of Twitter use. Articles in impactful journals can become heavily cited, but articles in journals with lower impact factors can generate considerable Twitter activity and also become heavily cited. Authors may benefit from establishing a strong social media presence, but should not expect research to become highly cited solely through social media promotion. Our research demonstrates that altmetrics and traditional metrics can be closely related, but not identical. We suggest that both altmetrics and traditional citation rates can be useful metrics of research impact.

## Introduction

Scientific writing is at the core of numerous professions, including academics, industry, government and agency work, and others. The success with which we often measure the breadth and impact of an individual’s written output forms the basis for job promotion, future research, products, and other important outputs. Accordingly, researchers are under constant pressure to boost traditional metrics of research output—namely the *h*-index, which accounts for an author’s number of publications (research output) and citation rates (research impact/quality). Therefore, an accurate understanding of the dynamics that make some scientific articles successful (where success is defined by future citations) is insightful for both individual scientists and research organizations.

Traditional metrics of citation rates have been criticized because, among other factors, they differ depending on the database (e.g. Web of Science, Scopus, Google Scholar) from which they are generated [1, 2]. Accordingly, in the past decade, researchers have sought alternative metrics of research impact (termed *altmetrics*) [3–5]. Commonly-used altmetrics include reads on reference managing websites such as Mendeley [6, 7], as well as mentions on blogs [8] or social media/micro-blogs (e.g. Facebook and Twitter) [9–11]. Some authors have suggested that altmetrics can be used as an alternative measure of research impact because they represent ‘real-time’ gauges for the amount of interest an article generates, and can sometimes predict whether or not an article will become highly cited [7, 12, 13]. However, many studies have shown low correlations between altmetrics and traditional citation rates [9, 14–16]. On the one hand, this suggests that altmetrics may be an imprecise measure of research impact. On the other hand, low correlations may indicate that altmetrics operate independently of traditional metrics, and should be considered as an alternative measure of research impact. Quantifying the precise relationship between altmetrics and traditional citation metrics is critical for resolving this issue.

Given the rise in popularity of altmetrics, scientists are increasingly using online social media platforms such as Twitter, Facebook, and ResearchGate to promote their research [17, 18]. Resources abound for researchers seeking to improve their scholarly impact via social media [19], and many journals now cultivate a strong online presence to broadcast their articles to a wide audience [16]. However, the actual benefit (in a traditional sense) to using social media for promoting scientific research remains poorly quantified. Accordingly, most academic institutions still consider only traditional metrics of scholarly output when evaluating researchers, and may even discourage social media use by employees as a waste of time. Understanding the relationship between altmetrics and traditional citation metrics is an important step toward modernizing our understanding of the true societal impact of scientific research. How does social media activity associated with scientific journal articles translate into more traditional metrics of scholarly output (i.e. citation rates)?

Unfortunately, statistical relationships between altmetrics and traditional citation metrics remain imprecisely quantified. Generalizable conclusions remain difficult to identify due to limited scope and/or methodological issues of various studies. For example, some studies have focused only on single journals [10, 13], or have had relatively small sample sizes [20]. Further, many studies have employed simple correlative analyses that cannot account for confounding factors that may also affect citation rates; these include journal impact factor and time since publication [8, 9, 16]. In this study, we examined the effects of Twitter activity on traditional citation rates of primary research articles in the recent ecological literature. We focused only on Twitter because (a) it constrains user interpretation to 140 characters of text, thus minimizing potential user bias, (b) previous research has shown Twitter to be the primary social media platform used by scientists for non-technical communication of their research, and (c) Twitter is correlated with activity on other social media platforms [10]. We modeled the number of citations on Thompson Reuters Web of Science^®^ database—often considered the ‘gold standard’ metric of traditional citation counts because of its reputation and stringent requirements for citations. We simultaneously controlled for the confounding effects of other important predictors, including journal impact factor, time since publication, and heterogeneity among journals. This approach provides estimates of the effects of Twitter activity on citation rates that are unbiased by many typical confounding factors. In taking an observational approach (as opposed to a manipulative experimental approach), we use the term, ‘predict’ in a statistical sense. That is, we did not ‘forecast’ the distribution of some unknown data based on some known relationship, but rather quantified relationships between predictor and dependent variables.

## Materials and Methods

We collected Twitter activity (defined here to include three metrics: number of tweets, number of users, twitter reach; see Table 1) and citation data on articles from twenty journals that publish only ecological research (Table 2). We excluded general scientific journal articles that include ecology as a disciplinary subset (e.g. *Science* or *Nature*), and ecology journals that publish only reviews and/or nontechnical pieces (e.g. *Frontiers in Ecology and the Environment* and *Trends in Ecology and Evolution*). We selected journals to represent a range of impact factors (IF, identified from Thompson-Reuters 2014 Journal Citation Reports^®^ database). We used 5-year IF as a metric of journal impact because it is more stable than yearly IFs, and is representative of most traditional measures of journal impact. We excluded journals with IF < 3.0 to minimize zero-inflation in the distribution of citation rates (i.e. many journal articles with zero citations). This cutoff value also serves to reduce potential unwanted variation caused by discipline-specific differences in Twitter activity: higher-impact ecology journals feature only general ecology, while discipline-specificity increases as impact factor decreases. Lower-impact journals typically focus more on specific processes, taxa, or systems, and are much more heterogeneous in many aspects of publication (e.g. article promotion, publication time, research timeliness, etc.). We acknowledge that this approach may bias some of our results by inflating the number of papers that have been mentioned on Twitter [21], or by missing a few key patterns within outlying journals. However, we are confident that our broad coverage of twenty journals and nearly 1,600 articles helps to ensure that we observed the true underlying patterns in the ecological research. We collected data on articles published from 2012 to 2014 to further minimize zero-inflation in citation distributions, as this allowed time for an article to be cited [22] and ensured articles published before the common use of Twitter were excluded. Because most social activity surrounding an articles occurs within a week of its initial publication, our time window also helped to reduce the possibility of Twitter use data changing dramatically throughout the data collection period.

**Table 1:** Definitions and summary statistics of variables used in generalized linear mixed models predicting citation rates of ecological research articles.

Metric	Definition	Average	Standard deviation	Median	Minimum	Maximum
Number of tweets	Number of individuals tweets and retweets associated with an article	5.7	14.2	2	0	343
Number of users	The number of individual Twitter accounts that sent tweets/retweets about an article	5.0	11.0	2	0	183
Twitter reach	The number of individual Twitter accounts that potentially viewed tweets associated with an article	14906	55096	2110	0	695914
Time since publication (days)	The number of days between the date on which an article was published and when citation data were collected	1089	337	1086	455	1904
Number of Web of Science citations	The number of articles indexed by Thompson-Reuters Web of Science that cite an article	11.9	12.9	8	0	154

**Table 2:** Impact factors, number of issues per year, and number of articles sampled from twenty ecological research journals.

Journal	5-year impact factor	Number of issues per year	Number of articles sampled
Animal Conservation	3.2	6	102
Conservation Letters	6.4	6	53
Diversity and Distributions	5.4	12	105
Ecological Applications	5.1	8	48
Ecology	6.2	12	108
Ecology Letters	16.7	12	106
Evolution	5.3	12	108
Evolutionary Applications	4.6	10	74
Fish and Fisheries	8.1	4	36
Functional Ecology	5.3	12	54
Global Change Biology	8.7	12	108
Global Ecology and Biogeography	7.2	12	108
Journal of Animal Ecology	5.3	6	54
Journal of Applied Ecology	5.9	6	54
Journal of Biogeography	4.6	12	108
Limnology and Oceanography	4.4	6	54
Mammal Review	4.3	4	31
Methods in Ecology and Evolution	7.4	12	90
Molecular Ecology Resources	4.9	6	54
New Phytologist	7.8	16	144

We randomly selected three articles from each issue, regardless of the number of articles per issue or the number of issues per year published by the journal. To avoid biased caused by article type, we collected data only on primary research articles, and excluded reviews, opinion pieces, and other non-technical articles. For example, essays and opinion pieces may be more heavily mentioned on social media platforms, while review papers are generally more heavily cited [21]. For each article, we first identified the number of citations the article had received on Thompson-Reuters Web of Science^™^ (WS) database. WS citations are more conservative than other citation metrics (e.g. Google Scholar), because WS only includes citations by articles in indexed, peer-reviewed journals [1, 2]. Time since publication is naturally indicative of citation rates; the longer a paper has been published, the longer it can accrue citations [23]. To calculate *time since publication*, we subtracted the Julian date (a unique ordinal integer assigned to dates) on which an article was published online from the date on which article-specific data were collected; this variable was measured in days. Exact dates of online publication are stated on each article. We were not interested in the effects of *time since publication per se*, but more so including it in the model to account for its confounding effects. Including such variables in models is necessary to pre-scale response variables, preventing these response variables from being nonsensical.

We then collected three metrics of Twitter activity, each of which were provided online for each journal article by Altmetrics, a company that gathers and summarizes coverage of scientific research articles in various media outlets (e.g. Twitter, Facebook, news outlets, etc.) [24]. Altmetrics tracks articles in real time through rich site summary (RSS) feeds, ensuring that data on Twitter activity are continuously up-to-date [25]. For each article, we collected: 1) the number of unique tweets about the article (hereafter, ‘*number of tweets*’), 2) the number of individual Twitter accounts (e.g., journal, author, other user) that sent those tweets (hereafter, ‘*number of users*’), and 3) *Twitter reach*, the number of individual Twitter accounts that potentially viewed tweets about an article (Table 1). All data were collected between 01 February and 05 April 2016.

Prior to analysis, we first screened predictor variables for multicollinearity and nonlinear (e.g. bimodal) relationships to WS citations using correlation matrices and bivariate plots. We found no evidence of bimodality, and thus included no quadratic terms in the model. If two variables had *r*>0.70, we removed one from analyses to reduce redundancy; we retained the one we believed to be more indicative of the underlying causal structure (e.g., *number of tweets* and *number of users*). We then standardized predictor variables to mean = 0 and variance = 1 to facilitate comparison of effect sizes, and to ensure maximum likelihood convergence. We used a generalized linear mixed model (GLMM) to identify the effects of metrics Twitter use on WS citation rates, while simultaneously accounting for the potentially confounding effects of journal IF and time since publication. We were not interested in identifying differences in citation rates among specific pairs of journals, but rather to generalize our findings from a random selection of journals to the overall population of ecological journals. Accordingly, we included journal identity as a random effect in the GLMM. This approach serves to account for nestedness of predictor variables (e.g. journal impact factor) within specific journals, and to account for inherent variability among journals that may affect WS citations (e.g. taxonomic bias, publisher quality, total number of articles published, or use of social media). Moreover, the random slopes better accommodate potential differences in bivariate relationship strength between predictor and dependent variables, rather than forcing them to a common average among journals. WS citation rates were modeled as a random variable of a negative binomial distribution [26]. Predictor variables were considered to have statistically significant effects if the 95% confidence intervals associated with their parameter estimates did not bound zero; this approach is analogous to a frequentist significance test at α = 0.05. We ran all analyses in R version 3.0.3 [27].

We used an information theoretic framework to compare the relative importance of Twitter activity, IF, and time since publication on WS citation rates. We used the *dredge* function in the *MuMin* package in R to calculate Akaike weights representing weight-of-evidence (*w*_*i*_) for GLMMs of all possible combinations of independent variables. For each variable, *w*_*i*_ was summed for all models containing that variable to calculate relative importance. Relative importance values range from 0 to 1, but do not sum to 1.0 among variables because multiple variables can be present in all models in the top model set (e.g. models with Σ*w*_i_ = 0.95). Variables with higher summed *w* values are considered more important [28]. Lastly, it is possible that citation rates are driven indirectly by IF via Twitter activity because higher-impact journals may be more widely-discussed by online users. We tested this hypothesis by using mixed effects models to estimate the effects of IF on *number of tweets* and *Twitter reach*, while including journal identity as a random effect.

## Results

We collected a total of 1,599 primary research articles among the twenty ecology journals over three years. Twitter activity and citation rates were variable among articles (Table 2). Over a fourth (28%, *n* = 442) of articles had no Twitter activity, and another 17% (266) had only one tweet. *Number of tweets* and *Twitter reach* were moderately correlated (*r* = 0.65); *Twitter reach* of single-tweet articles ranged from 0 (one tweet from an account with zero followers) to 10,939 users. However, *number of users* was strongly correlated with *number of tweets* (*r* = 0.97); we thus excluded *number of users* from GLMM analysis.

**Fig 1:**
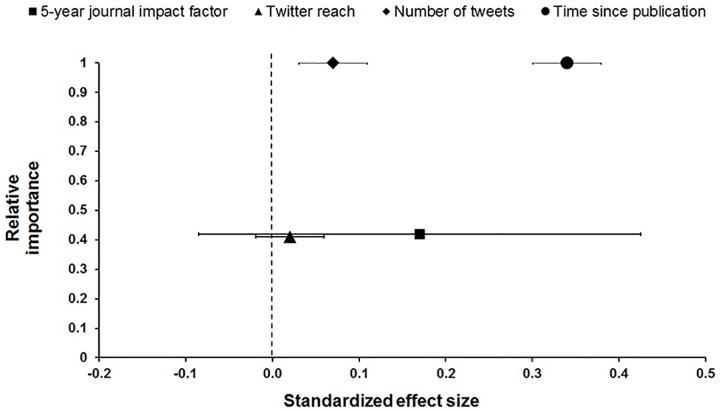
Relative importance (vertical) and standardized effect size (±95% confidence intervals) (horizontal) of 5-year journal impact factor, Twitter reach, number of tweets, and time since publication in generalized linear mixed models predicting the number of Web of Science citations of 1,599 primary ecological research articles published in 20 journals between 2012 and 2014. Note that relative importance of number of tweets is also 1.0, but is offset to display confidence intervals.

**Fig 2:**
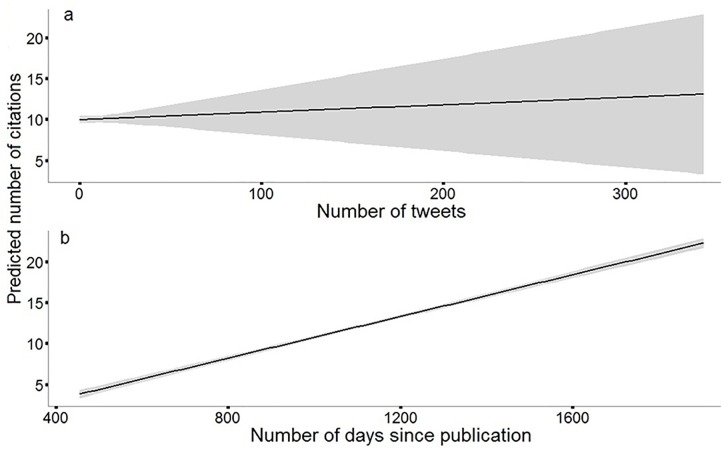
Model-predicted number of Web of Science citations an article received as a function of (a) the number of tweets about that article and (b) time since the article was published. The solid black line represents a fitted line from predicted values bounded by standard error (gray).

Twitter activity was a significant positive predictor of citation rates. *Number of tweets* and *time since publication* were significantly related to the number of Web of Science citations articles received. Parameter estimates for these variables had 95% confidence intervals that did not bound zero (equating to *p*<<0.05). However, the effects of *number of tweets* was approximately one-fifth as strong as *time since publication*. The effects of *5-year IF* and *Twitter reach* did not significantly influence citation rates; these parameters had 95% confidence intervals that bounded zero (Fig 1). With relative importance values of 1.0, *time since publication* and *number of tweets* were the most important predictors of WOS citations; Journal *5-year IF* and *Twitter reach* had relative importance of 0.42 and 0.40, respectively (Fig 1, Table 3). Fig 2 displays model-predicted values (± as a function of actual (unscaled) predictor variables that had significant effects on the number of WOS citations.

**Table 3:** Standardized effect sizes (±standard error) of variables affecting the number of Web of Science citations received by 1,599 primary research articles published in ecology journals between 2012 and 2014.

Independent variable	Parameter estimate (±standard error)	Relative importance
5-year journal impact factor	0.17±0.13	0.42
Number of tweets	0.07±0.02	1.00
Twitter reach	0.02±0.02	0.41
Time since publication	0.39±0.02	1.00

Two lines of evidence demonstrate that Twitter activity was not driven by journal impact factor. First, *5-year IF* was very weakly correlated with both *number of tweets* and *Twitter reach* (*r* = 0.11 for both). Second, *5-year IF* did not have significant effects on *number of tweets* or *Twitter reach* (*b* = 0.15±0.18 and *b* = 0.34±0.34). These results do not support the hypothesis that positive relationships between Twitter use and citation rates exist because higher-impact journals are more popular and thus receive greater social media attention within the set of journals we examined.

## Discussion

This study provides evidence that Twitter activity associated with primary ecological research articles is significantly and positively associated with the number of future citations. Although focusing solely on higher-impact ecology journals, this represents the first study to compare the relative effects of social media activity, journal impact factor, and time since publication on citation rates of research from any discipline. In doing so, we found that the role of journal IF can be strong but variable, and that the effect of time since publication can outweigh both Twitter activity and IF. Our inclusion of multiple journals demonstrates that these patterns are not specific to any one particular journal, but instead are generalizable across journals within the discipline of ecology. Although inference may be constrained within the discipline of ecology, we expect the patterns to hold across other disciplines, as well [14, 29].

Moving beyond simple correlative approaches is necessary for parsing out the relationship between social media activity and other factors on traditional measures of scholarly impact [22]. Past studies have provided rather ambiguous inference, showing weak (and even negative) correlations between Twitter activity and citation rates. For example, Thelwall et al. [15] found a correlation of -0.19 between Twitter use and citation rates, Haustein et al. [16] found *r* = 0.10–0.18, Priem et al. [9] found *r* = 0.10, and Costas et al. [14] found *r* = 0.14–0.22. In fact, simple log-transformed correlations reveal similarly weak relationships between WS citations and *number of tweets* (*r* = -0.05) and *Twitter reach* (*r* = -0.06) in our dataset. On the surface, our results would fit in line with the aforementioned studies. However, accounting for time since publication and journal identity in the same statistical model was critical, and not only made our model a more accurate representation of the process we were trying to describe, but revealed significant positive effects of Twitter activity on citation rates. Future work accounting for other potential confounding factors such as number of authors, article length/type, and open access policies [21, 30], will refine our understanding of how altmetrics relate to traditional citation metrics.

A few studies have sought to control for time since publication when analyzing the relationship between social media use and citation rates. Thelwall et al. [15] introduced a ‘sign test’, which categorically compares the number of citations a paper receives to those published just before and just afterward. While this can be a useful approach, interpretation may be limited because observations are not independent of one another. In analyses somewhat similar to ours, Eysenbach [13] and de Winter [10] used multiple linear regression to parse out the effects of time since publication and various metrics of Twitter use and citations. Our results are similar to de Winter’s [10], who found that the effect of Twitter activity was about a third as strong as time since publication. Like time since publication, accounting for random variability among journals is critical for estimating the true effects of interest. Mixed effects modeling is an ideal tool for statistically comparing the relative effects of multiple variables on a response of interest, and will be useful for researchers continuing to investigate the effects of social media use on research citation rates.

Another key finding of this study is that *number of tweets* had more precise effects on citation rates than journal impact factor for the suite of journals we examined. Although the effect size of *5-year IF* was approximately twice that of both metrics of Twitter use, it was unreliably imprecise (i.e. not statistically significant). Moreover, the weight-of-evidence (relative importance) of IF was less than half of *time since publication* and *number of tweets*. Journal IFs as a measure of research importance have been criticized for being weakly correlated with citation rates of individual articles. For a given journal, a few articles may become heavily-cited, while most have more modest citation rates [31, 32]. As a result, some researchers are suggesting that altmetrics can be an complimentary currency of initial research impact [33, 34]. However, Branch et al. [30] showed that articles in very high-impact journals (i.e. *Science*, *Nature*, *and Proceedings of the National Academy of Sciences*) receive many more citations than articles on lower-impact journals. Accordingly, we recommend that interpretations be made within the context of the discipline, journals, and impact factors we examined. The true effect of journal impact factor may be more apparent across a wider range of journals and disciplines.

Some researchers have interpreted weak correlations between altmetrics and traditional citation metrics as evidence that altmetrics capture an inherent property of scientific research that is unquantifiable by traditional citation metrics [9, 14, 16], and thus are preferable (or at least independent) to traditional citation metrics. On the one hand, our study supports that hypothesis in that altmetrics can be more precise statistical predictors of citations than traditional metrics such as journal impact factor among higher-impact ecology journals. This interpretation is particularly intuitive, as the altmetrics we used are measured at the resolution of individual papers (finer scale), while impact factor was measured at the resolution of journals (coarser scale). Further, we show that Twitter activity associated with primary ecological research was mostly independent of journal impact factor; ecology journals with higher IF were not necessarily the most discussed on Twitter. On the other hand, however, *number of tweets* was significantly related to traditional citation rates. This suggests that altmetrics and traditional metrics may not be more closely associated than previously believed. It is possible that the ‘signal’ of their relationship with traditional metrics have been masked by the ‘noise’ of confounding factors such as time since publication and random journal variability. We conclude that altmetrics can be useful measures of research impact, but a role still exists for traditional citation rates within the discipline of ecology.

The strong relationships we found between Twitter activity and traditional citations are certainly predictive (in a statistical sense), but not necessarily causal. That is, we do not interpret our results as suggesting that low-quality ecology research will become heavily-cited simply because it is highly-tweeted. Alternatively, our results do suggest that ecological researchers should not expect a paper to become highly-cited simply because it is published in a high-profile journal. Eysenbach [13] warned that researchers could ‘game the system’ by unscrupulously over-broadcasting links to their research on social media, leading to a type of ‘pathological publishing’ [35]. However, our application of two statistically independent metrics of Twitter activity (*number of tweets* and *Twitter reach*) suggests otherwise. Even if researchers took this approach, the effect on their traditional citation rates would be limited by their number of followers on social media because the *number of tweets* and *Twitter reach* were not strongly correlated. Although this situation may be possible from a user with many followers, our results suggest that the true power of numbers lies more in the collective online community, and less on the individual user.

Within the scope of the journals and discipline we examined, our results suggest that quality ecological research will be discussed on Twitter and cited, regardless of journal impact factor. Researchers should therefore benefit from cultivating a strong online presence *and* publishing high-quality research. Because social technology algorithms are designed to display content of higher relevance to each user, active users on Twitter are more likely to see content that is viewed and shared more often by people within their social technology networks (i.e. ecologists tweeting to ecologists). Our results suggest that Twitter can also function as an alternative discovery mechanism for scientific articles that is 1) dependent on each article's value and relevance to colleagues within social networks and 2) independent of the distribution and popularity of each journal. Accordingly, it is possible that our selection of generally more popular (e.g. higher impact) ecological journals may have a bearing on the patterns we observed. It is also entirely possible that certain readership thresholds exist, below which papers are less likely to be cited without online promotion simply due to low journal readership and/or high specificity, regardless of the quality of the research; we acknowledge this issue with our analysis. A logical next step will be to research the effect of social media use among journals with lower impact factors to determine if Twitter activity can boost citation rates by increasing readership of individual articles. More research is also needed across disciplines to determine the true relationship between altmetrics and traditional metrics of scholarly output of scientific research.

## Supporting Information

S1 Dataset Data used in this study. Variables include *journal identity*, *5-year journal impact factor*, publication information (*year published*, *volume*, *issue*, and *authors*), *collection date* and *publication date* (used to calculate *time since publication*), *number of tweets*, *number of users*, *Twitter reach*, and *number of Web of Science citations*.
(CSV)
